# Burden of non-invasive fungal infections and antifungal therapy in pediatric primary care settings: FUNGICARE Project

**DOI:** 10.1186/s13052-026-02277-8

**Published:** 2026-05-26

**Authors:** Lorenzo Chiusaroli, Emelyne Gres, Claudia Cozzolino Cangiano, Anna Cantarutti, Daniele Donà, Carlo Giaquinto, Vincenzo Baldo, Elisa Barbieri

**Affiliations:** 1 Division of Pediatric Infectious Diseases, Department for Women’s and Children’s Health, University of Padua, 35126 Padua, Italy; 2 Department of Molecular Medicine, University of Padua, Padua, Italy; 3 Department of Cardiac, Thoracic, Vascular Sciences and Public Health, University of Padua, 35126 Padua, Italy; 4 Unit of Biostatistics, Epidemiology and Public Health, Department of Statistics and Quantitative Methods, University of Milano-Bicocca, 20126 Milan, Italy; 5 National Centre for Healthcare Research and Pharmacoepidemiology, Department of Statistics and Quantitative Methods, University of Milano- Bicocca, 20126 Milan, Italy; 6 Preventive Medicine and Risk Assessment Unit, Hospital-University of Padua, 35126 Padua, Italy

**Keywords:** Fungal infections, Pediatric, Children, Epidemiology, Antifungal treatment, Primary care, Outpatients

## Abstract

**Background:**

Fungal infections are common worldwide and a frequent reason for pediatric primary care visits, mainly affecting the skin, hair, and nails, with a consistently high prevalence in outpatient settings. This study aimed to assess the burden of fungal infections in pediatric primary care in Italy and characterize the patterns of antifungal therapy use.

**Methods:**

This population database analysis used data from Pedianet, a comprehensive database of 193 family pediatricians in Italy. The annual incidence rate (IR) of fungal infections was evaluated in children aged < 14 years from January 2010 to December 2024. Subjects were followed up from 2010 or their enrollment date until the end of assistance or the end of the study period. All antifungal treatments occurring within an episode were included.

**Results:**

A total of 23,463 episodes of fungal infection were identified, including 23,034 episodes of skin and mucocutaneous mycoses and 429 episodes of onychomycosis. The most frequent diagnoses were *Candida*-associated diaper dermatitis (52.1%) and mucocutaneous candidiasis (27.4%). Between 2010 and 2019, the incidence of fungal infections remained stable at 11–14 cases per 1,000 person-years. A marked decline across all age groups was observed in 2020, reflecting the impact of the SARS-CoV-2 pandemic. Regarding treatment, 144/429 (33.6%) of onychomycosis episodes received an antifungal prescription, mainly tioconazole, while 8347/23,034 (36.3%) of skin and mucocutaneous mycoses were treated, predominantly with clotrimazole.

**Conclusion:**

This population-based cohort study updates the epidemiology of non-invasive fungal infections in pediatric outpatients and underscores the need for greater awareness, improved diagnosis, prevention, and antifungal stewardship in primary care.

**Supplementary Information:**

The online version contains supplementary material available at 10.1186/s13052-026-02277-8.

## Background

Fungal infections (FIs) are ubiquitous and are increasingly recognized by the World Health Organization (WHO) as a major public health concern [1]. According to data from the Centers for Disease Control and Prevention (CDC), fungal diseases in the United States alone are associated with approximately 13 million outpatient visits annually, over 130 000 hospitalizations, and more than 7 000 deaths each year [2]. Globally, superficial fungal infections affecting skin, nails, hair, and mucous membranes affect an estimated 20–25% of the population at any one time, and the burden is rising, with a global estimate of 1.73 billion incident cases of fungal skin diseases in 2021 [3, 4].

In pediatric populations, FIs are frequently encountered in outpatient and primary care settings, particularly involving keratinized tissues and mucosal surfaces. Dermatophyte infections (*tinea corporis*, *tinea capitis*, onychomycosis) are common among children, and non-invasive *Candida* infections, such as oral thrush, diaper-area candidiasis, and vulvovaginal candidiasis (VVC) in adolescents, also contribute substantially to clinical workload [5].

These infections, though typically considered mild and manageable, warrant attention because they may serve as reservoirs for colonization and, under certain circumstances, may precede invasive disease. In this context, in neonates and preterm infants, mucocutaneous involvement with *Candida* spp. (such as thrush or diaper dermatitis) has been identified as a potential harbinger of systemic infection [6].

Therapeutic strategies for fungal infections have evolved over recent decades. The introduction of newer antifungal agents and formulations (such as echinocandins, liposomal amphotericin B, and second-generation azoles) has expanded treatment options and improved outcomes for many patients [7, 8]. At the same time, superficial fungal infections are often underdiagnosed or misclassified, due in part to variable clinical presentations and limited access to diagnostic mycology, even though modern molecular techniques are increasingly integrated into clinical practice [9].

This study aimed to investigate the burden and incidence of FIs in Italian children and to characterize the use and treatment with antifungal therapies in primary care.

## Methods

### Data sources

The Pedianet database was used as the source of the study. Pedianet is a national population database that contains anonymous patient-level data of more than 500,000 children since 2004, corresponding to around 4% of the annual pediatric population, who received healthcare from over 250 family pediatricians (FPs) in Italy who were part of the Pedianet^®^ network [10].

For this study, we included the data of 193 FPs who contributed to the database from 2010 to 2024; the data were extracted on 23 February 2025. According to the Italian National Health Service (NHS), each child is assigned to a FP who is the primary referral for health-related matters. In Italy, there is a tax-funded public healthcare system with universal access, and patients incur no direct costs for primary care visits. The Pedianet database captures several types of patient-level information, including the reason for accessing healthcare, health status, demographic data, diagnosis, and clinical symptoms (free text or International Classification of Diseases, 9th Revision, Clinical Modification, ICD-9-CM, codes), drugs (Anatomical-Therapeutical-Chemical, ATC, codes), specialist appointments, diagnostic procedures, hospital or emergency room admissions, growth parameters, and clinical outcome data. Data are anonymized and updated monthly in a centralized database hosted by So.Se.Te., the legal owner of Pedianet^®^, in Padova. Informed consent from the children’s parents is required for data inclusion in the database. For participants who provide consent, the electronic health records are enriched with information from additional data sources, including vaccination registry and hospitalization records, using unique patient identifiers.

Data are manually validated for study-specific conditions, and the accuracy of the diagnosis data was verified.

### Study design and population

This population database analysis included all children aged 0–14 years enrolled in the Pedianet database from January 1, 2010, to December 31, 2024. For each participant, follow-up began on the 15th day of the birth month or the date of enrolment, whichever occurred later. Follow-up ended at the earliest of the end-of-care date, or December 31, 2024. Children older than 14 years, those with insufficient follow-up for the identified FIs, or those with missing information on sex or month of birth were excluded from the analysis.

### Non-invasive fungal infection episode definition

Non-invasive FIs were identified using records from primary care visits, emergency department visits, and hospitalizations reported by primary care pediatricians. Diagnoses were identified using ICD-9-CM codes and free-text entries in medical notes, which were manually validated by a clinician. We distinguished skin and mucocutaneous mycoses from onychomycosis based on differences in typical treatment duration. An episode of skin or mucocutaneous mycosis included all infection-related records occurring within two months of each other and up to two months after the last record [11]. An episode of onychomycosis included all infection-related records occurring within 1 year of each other and up to 1 year after the last record [12].

Recurrence was defined as one episode occurring after the end of the 2-month follow-up period for skin or mucocutaneous mycosis and after the end of the one-year follow-up period for onychomycosis.

Given the current geographic epidemiology, we did not include endemic mycoses or zoonotic dermatophytosis, as they are not widespread in Italy.

### Outcomes

All prescriptions, identified using ATC codes, issued during the FIs episode were considered part of the treatment for that episode. Topical and systemic antimicrobial exposure was defined based on prescriptions for medications classified under ATC codes starting with D01, D06, and D07 for topical agents, and J02 for systemic agents. When two or more prescriptions with the same specific 7-digit ATC code were recorded during the same visit for the same diagnosis, they were counted as a single prescription.

Treatment failure was defined as the prescription for a second antifungal, either topical or systemic, within 60 days for non-invasive FIs. For onychomycosis, a second prescription within one year of follow-up was considered indicative of treatment failure. Adverse events (AE) occurring during the episode were also identified with ICD-9-CM codes and free text (Table S1).

### Covariates

The baseline characteristics of the study included sociodemographic factors like age, sex, and geographic location (North, Center, South with Islands). Age was stratified into the following categories: <2 months, 2–12 months, 13 months–4 years, 5–9 years, 10–14 years. We also included comorbidities, chronic conditions such as respiratory issues (like asthma, bronchopulmonary dysplasia, and wheezing), cardiovascular problems, diabetes, immunosuppression, or immunosuppressive therapy (including cancer therapies), Down syndrome, and dermatological problems (like psoriasis, atopic dermatitis, chronic urticaria, alopecia areata, and vitiligo).

### Statistical analysis

Descriptive analyses were summarized using tables and graphical representations, reporting means, medians, and standard deviations for continuous variables, and frequency distributions for categorical variables. The incidence rate of FIs was calculated by dividing the number of episodes during follow-up by the total person-time, expressed in years from start to end of follow-up, and reported per 1,000 person-years. The person-time was calculated by splitting each patient’s follow-up period (from the date of cohort entry to the end of follow-up or December 31, 2024) into monthly intervals, then summing the total number of follow-up months (converted into person-years), stratified by calendar year and age group. 95% confidence intervals (CIs) were also computed. Annual incidence rates were described for the overall cohort and stratified by age groups.

Trends for fungal infection incidence (per 1,000 person-years) were assessed using joinpoint regression analysis. An algorithm tests whether trends in annual percentage change (APC) of incidence fit a series of joined straight lines on a logarithmic scale. The line segments converge at specific points, called joinpoints, which indicate a change in trend. The weighted Bayesian Information Criterion (BIC) method was used to identify the number of significant joinpoints. Using the calendar year as a variable, the joinpoint regression analysis estimated the APC of the rates between each joinpoint and the average annual percentage change (AAPC) with a 95% confidence interval (CI). We estimated the autocorrelation of our data using an autocorrelogram included in the model. The jointpoint regression analyses were performed using the Joinpoint software version 5.4, May 2023 (Division of Cancer Control and Population Sciences, National Cancer Institute) [13].

All analyses were performed using R statistical software, version 4.0.3 (Vienna, Austria).

## Results

**Fig. 1:**
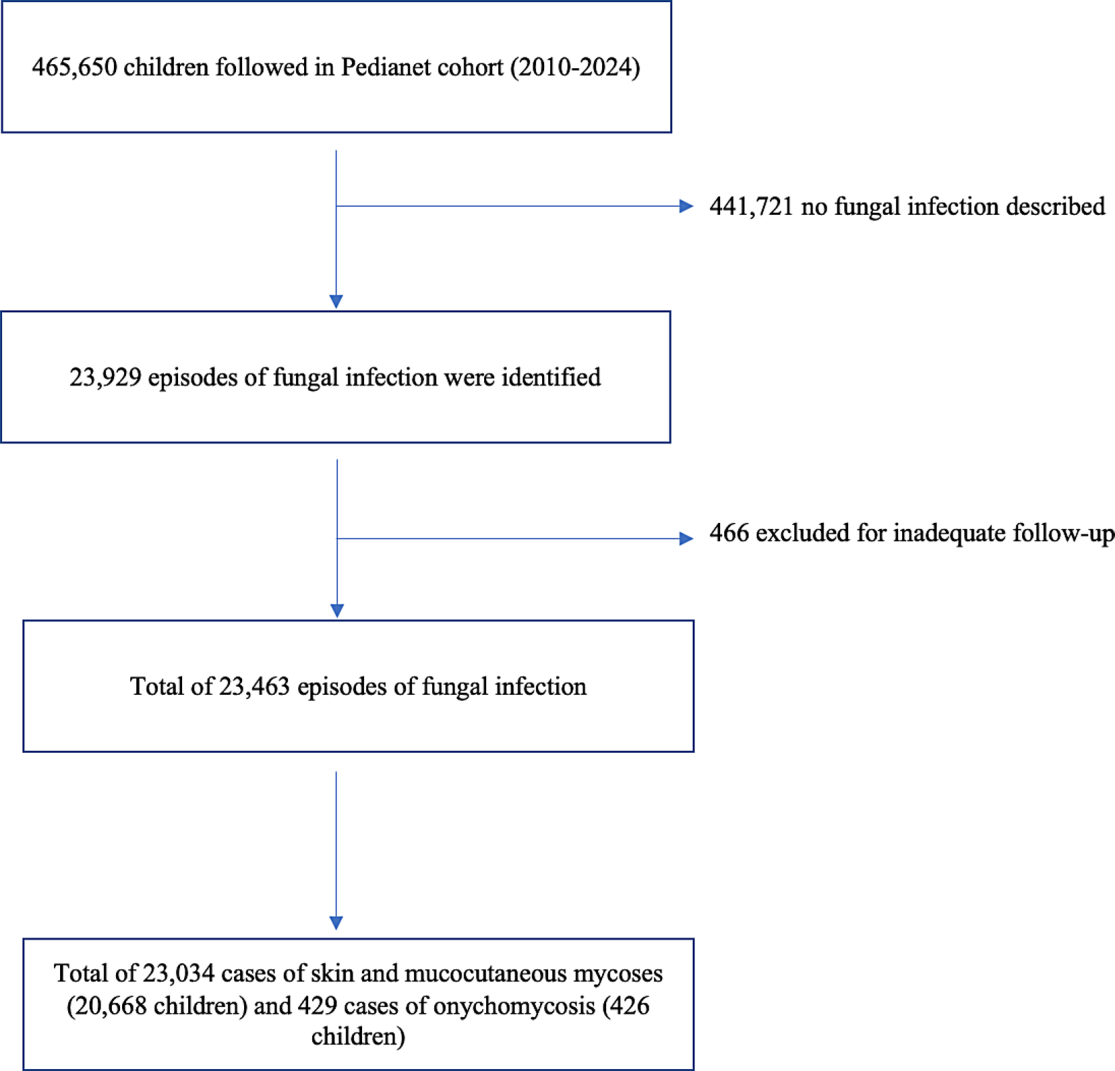
Flowchart of the study. Pedianet, 2010–2024

**Fig. 2:**
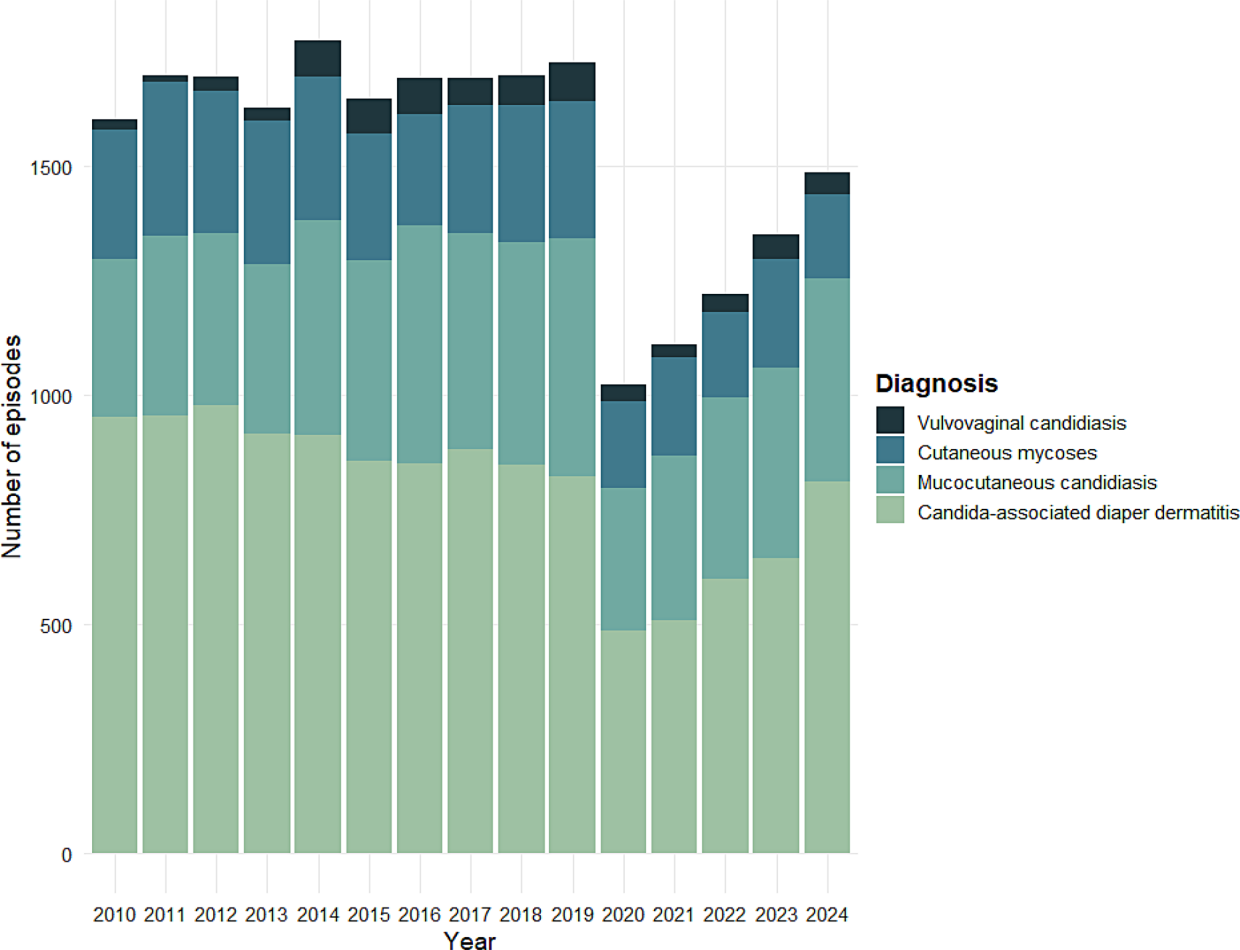
Yearly number of fungal infection episodes stratified by type of diagnosis. (Pedianet 2010–2024)

A total of 465,650 children were followed during the study period. Overall, 23,463 episodes of FIs were identified in 20,668 children, including 23,034 episodes (98.2%) of skin and mucocutaneous mycoses occurring in 20,281 children and 429 cases (1.8%) of onychomycosis occurring in 426 children (Fig. 1). Among skin and mucocutaneous mycoses, the most frequent diagnoses were *Candida*-associated diaper dermatitis (12,004/23,034; 52.1%) consistently accounted for the largest proportion of episodes throughout the study period, followed by mucocutaneous candidiasis (6,322/23,034; 27.4%), cutaneous mycoses (3,959/23,034; 17.2%), and vulvovaginal candidiasis (749/23,034; 3.3%) (Fig. 2).

**Fig. 3:**
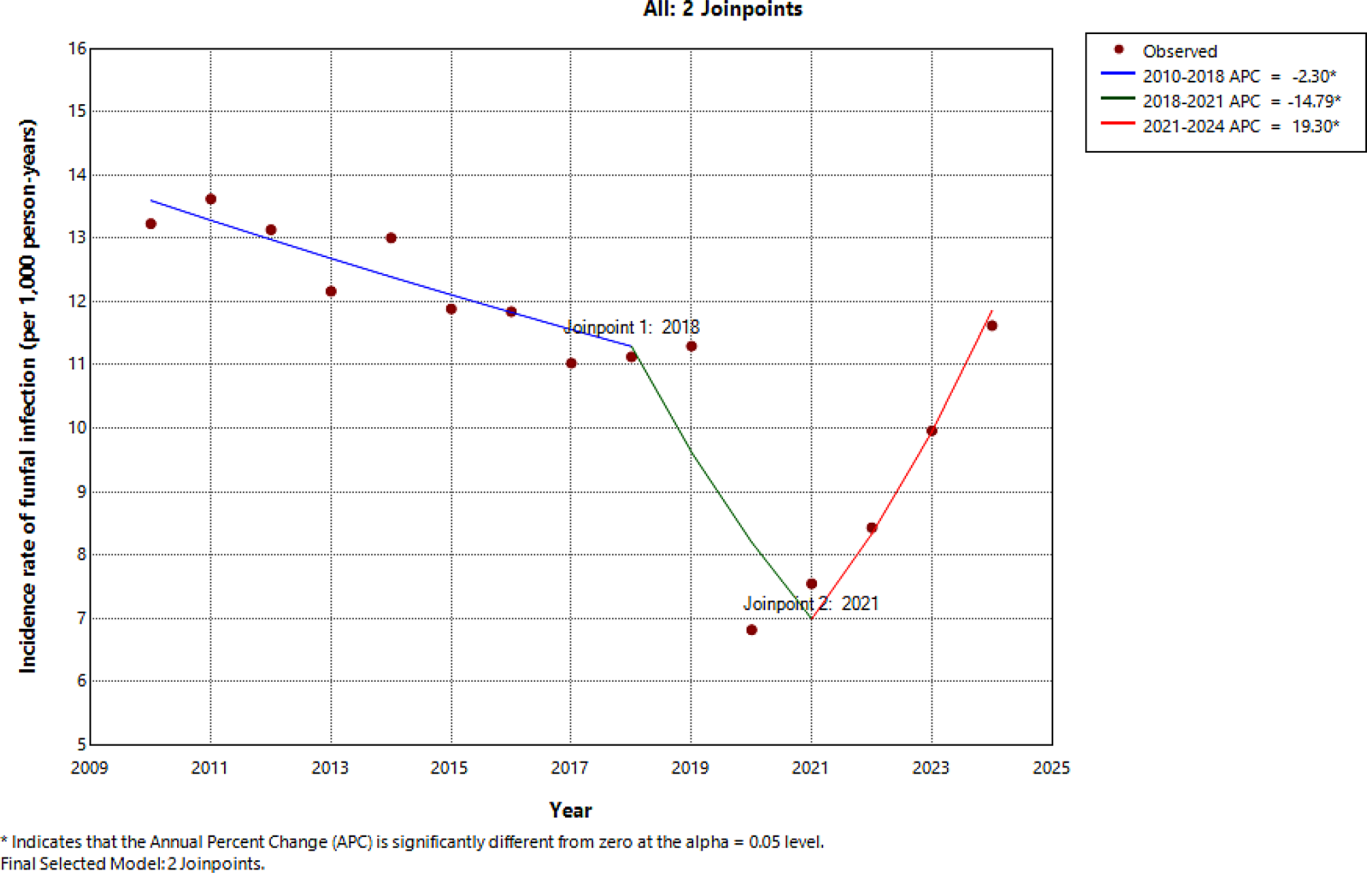
Temporal trends in the incidence of fungal infections and joinpoint regression analysis, 2010–2024

The overall incidence of FIs (skin and mucocutaneous mycoses and onychomycosis), described in Fig. 3. Joinpoint regression analysis identified two significant inflection points in 2018 and 2021. Between 2010 and 2018, the incidence rate of fungal infection showed a significant but modest decline (APC − 2.30%, 95%CI − 3.72 to − 0.94; *p* = 0.002). The decline became substantially steeper between 2018 and 2021, with an annual decrease of 14.79% (95% CI − 17.13 to − 9.86; *p* < 0.001). From 2021 to 2024, the trend reversed, with a significant increase in incidence (APC 19.30%, 95% CI 14.28 to 32.84; *p* < 0.000001). Overall, across the 2010–2024 study period, the incidence rate showed a small but significant downward trend, with an average annual percent change (AAPC) of − 0.97% (95% CI − 1.70 to − 0.28; *p* = 0.012).

Moreover, incidence varied considerably by age (Fig. S3). It was highest among children under 1 year, reaching approximately 80 cases per 1,000 person-years in those < 2 months and around 60 cases per 1,000 person-years in those aged 2–12 months during the study period. After the first year of life, incidence dropped below 20 cases per 1,000 person-years. A pronounced decline during the COVID-19 period was observed for the 2–12 months, 13 months–4 years, and 5–9 years age groups, with non-overlapping confidence intervals, likely reflecting enhanced hygiene measures and reduced social contact during this period (Supplementary Material Figure S3).

The sociodemographic characteristics and comorbidities of children at first presentation are presented in Table 1. Overall, females accounted for 54.8% of cases. Stratifying the cases by age, the highest burden was observed among infants aged 1 month to 1 year (7,474 episodes; 35.4%) and children aged 1–4 years (7,836 episodes; 37.1%) and a very low number is represented in adolescents (75; 0.4%). Most cases were reported by pediatricians in northern Italy, accounting for 58.0% of onychomycosis and 68.6% of skin and mucocutaneous mycoses. Regarding the features of the population, only the 5.2% of patients presented at least one chronic disease including prematurity (2.7%), asthma (0.9%), chronic neurological diseases (0.7%), and chronic endocrine disease (0.6%).

**Table 1:** Clinical and sociodemographic characteristics of the included cohort at the first episode of fungal infection

	Overall *N* = 21,094	Onychomycosis *N* = 426	Skin and mucocutaneous mycoses *N* = 20,668
**Sex**			
Female	11,561 (54.8)	201 (47.2)	11,360 (55.0)
Male	9,533 (45.2)	225 (52.8)	9,308 (45.0)
**Diagnosis**			
*Candida*-associated diaper dermatitis			12,004 (52.1)
Mucocutaneous candidiasis*			6,322 (27.4)
Cutaneous mycoses°			3,959 (17.2)
Vulvovaginal candidiasis			749 (3.3)
**Age**			
Mean (SD) - Median (Q1-Q3) - months	27 (38) − 12 (5–25)	78 (49) − 66 (36–124)	26 (37) − 12 (5–23)
< 2 months	2,912 (13.8)	1 (0.2)	2,911 (14.1)
2–12 months	7,474 (35.4)	9 (2.1)	7,465 (36.1)
13 m-4.99y	7,836 (37.1)	183 (43.0)	7,653 (37.0)
5-9.99y	1,578 (7.5)	120 (28.2)	1,458 (7.1)
10-13.99y	1,219 (5.8)	113 (26.5)	1,106 (5.4)
**Region**			
South + Island	4,558 (21.6)	137 (32.2)	4,421 (21.4)
North	14,423 (68.4)	247 (58.0)	14,176 (68.6)
Center	2,113 (10.0)	42 (9.9)	2,071 (10.0)
**Comorbidities**			
Overall	1092 (5.2)	42 (10)	995 (4.8)
Asthma	189 (0.9)	11 (2.6)	182 (0.9)
Autoimmune diseases	115 (0.5)	3 (0.7)	112 (0.5)
Blood diseases	25 (0.1)	3 (0.7)	22 (0.1)
Cancers	32 (0.2)	-	32 (0.2)
Cardiovascular diseases	110 (0.5)	8 (1.9)	103 (0.5)
Endocrine diseases	136 (0.6)	4 (0.9)	133 (0.6)
Gastroenterological diseases	8 (0)	-	8 (0)
Hepatic diseases	4 (0)	1 (0.2)	3 (0)
Immunodeficiency	6 (0)	-	6 (0)
Metabolic diseases	40 (0.2)	3 (0.7)	37 (0.2)
Multimorbidity	219 (1)	4 (0.9)	215 (1)
Neurological diseases	139 (0.7)	5 (1.2)	134 (0.6)
Organ transplant	7 (0)	-	7 (0)
Osteoarticular diseases	1 (0)	-	1 (0)
Other not specified comorbidities	61 (0.3)	2 (0.5)	59 (0.3)
Other respiratory diseases	11 (0.1)	-	11 (0.1)
Prematurity	579 (2.7)	6 (1.4)	573 (2.8)
Renal diseases	15 (0.1)	1 (0.2)	14 (0.1)

SD: Standard deviation

*We included in this definition episodes with oral thrush and episodes combined of oral thrush and fungal cutaneous infections

°We included in this definition episodes with *tinea capitis*, *corporis*, *pedis*, *Pityriasis versicolor* infections and, dermatomycosis

Among the 23,463 FIs episodes, 11,969 (51.0%) received treatment, and 8,491 (36.2%) received an antifungal treatment: 8,347 (36.3%) for skin and mucocutaneous mycoses and 144 (33.6%) for onychomycosis. Most patients received topical antifungal agents (8,729; 94.7%), and a minority received systemic agents (485; 5.3%). Clotrimazole was the most prescribed topical antifungal compound with 4,104 (44.5%) prescriptions, followed by miconazole 1,255 (13.6%), imidazole or triazole in combination with corticosteroids 816 (8.9%), and econazole 696 (7.6%). Among systemic antifungal prescriptions, the most prescribed medications were griseofulvin (235; 2.6%), terbinafine (71; 0.8%), and itraconazole (41; 0.4%). The completed findings of antifungal prescriptions are reported in Table 2.

**Table 2:** Treatment prescribed for the different episodes. Pedianet 2010–2024

	Overall	Onychomycosis	Skin and mucocutaneous mycoses
**Number of episodes**	23,463	429	23,034
Number of episodes without a prescription	11,494 (49.0)	130 (30.3)	11,364 (49.3)
Number of episodes with a prescription	11,969 (51.0)	299 (69.7)	11,670 (50.7)
**Number of treatments prescribed**	17,495	700	16,795
** Antifungal compounds**	9,214	166	9,048
Clotrimazole	4,104 (44.5)	27 (16.3)	4,077 (45.1)
Miconazole	1,255 (13.6)	20 (12.0)	1,235 (13.6)
Midazoles/triazoles, combination with corticosteroids	816 (8.9)	1 (0.6)	815 (9.0)
Econazole	696 (7.6)	11 (6.6)	685 (7.6)
Tioconazole	637 (6.9)	61 (36.7)	576 (6.4)
Fluconazole (topic)	443 (4.8)	6 (3.6)	437 (4.8)
Bifonazole	300 (3.3)	2 (1.2)	298 (3.3)
Griseofulvin	235 (2.6)	6 (3.6)	229 (2.5)
Ciclopirox	147 (1.6)	12 (7.2)	135 (1.5)
Ketoconazole (topic)	140 (1.5)	1 (0.6)	139 (1.5)
Terbinafine (topic)	110 (1.2)	0 (0.0)	110 (1.2)
Amorolfine	2 (0.0)	2 (1.2)	0 (0.0)
Fenticonazole	73 (0.8)	0 (0.0)	73 (0.8)
Fluconazole (systemic)	6 (0.1)	1 (0.6)	5 (0.1)
Flutrimazole	1 (0.0)	0 (0.0)	1 (0.0)
Isoconazole	96 (1.0)	0 (0.0)	96 (1.1)
Itraconazole	41 (0.4)	3 (1.8)	38 (0.4)
Ketoconazole (systemic)	1 (0.0)	0 (0.0)	1 (0.0)
Methylrosaniline	4 (0.0)	0 (0.0)	4 (0.0)
Naftifine	23 (0.2)	0 (0.0)	23 (0.3)
Sertaconazole	13 (0.1)	0 (0.0)	13 (0.1)
Terbinafine (systemic)	71 (0.8)	13 (7.8)	58 (0.6)
* Route of administration*			
Systemic	485 (5.3)	9 (5.4)	476 (5.3)
Topic	8,729 (94.7)	157 (94.6)	8,572 (94.7)
** Other associated treatment**	8,281	534	7,747
Antibacterial for systemic use	3 (0.0)	0 (0.0)	3 (0.0)
Antibacterial for topical use	435 (5.3)	19 (3.6)	416 (5.4)
Antiseptics and disinfectants	382 (4.6)	7 (1.3)	375 (4.8)
Beta-lactam antibacterial	4,664 (56.3)	357 (66.9)	4,307 (55.6)
Corticoids, combinations with antibiotics	540 (6.5)	18 (3.4)	522 (6.7)
Corticosteroids, dermatological preparations	642 (7.8)	14 (2.6)	628 (8.1)
Macrolides, lincosamides and streptogramins	1,019 (12.3)	91 (17.0)	928 (12.0)
Other antibacterial	287 (3.5)	7 (1.3)	280 (3.6)
Preparations for treatment of wounds and ulcers	141 (1.7)	5 (0.9)	136 (1.8)
Sulfonamides and trimethoprim	35 (0.4)	3 (0.6)	32 (0.4)
ATC code missing	43 (0.5)	2 (0.2)	41 (0.5)
Others	90 (1.0)	11 (2.2)	79 (1.0)

ATC: Anatomical Therapeutic Code

Finally, considering the treatment outcome for skin and mucocutaneous mycoses 1,086 (9.3%) episodes received more than one prescription at the first consultation, and the treatment was prolonged in 724 (6.2%) episodes (Supplementary materials Table S2). At last, an antifungal switch, considered a treatment failure, was reported in 91 (0.7%) episodes, with a median switch time of 22 days (Supplementary materials Table S2 and Figure S4). In onychomycosis, 21 episodes (7.0%) received more than one prescription at the first consultation, and treatment was prolonged in 72 (24.2%) episodes. Only three patients experienced treatment failure and required a switch to other antifungal agents (Supplementary materials Table S2 and Figure S4).

In our population, we reported a total number of 16 AEs during the observation period: 9 in male and 7 in female patients, and mainly represented in patients with 13 months-4 years old with 10/16 AEs, 2–12 months with 4/16 AEs, 5-9y with 2/16 AEs. However, all these patients had received other medications during the follow-up period, and only two presented AE directly correlated with miconazole therapy: one presented a skin rash concerning for allergy, and one presented a sensation of burning tongue.

## Discussion

Our study is a large cohort of FIs occurring in pediatric primary care, reporting the epidemiology, geographic distribution, age distribution, and treatment-associated outcomes.

Overall, there is very limited evidence on the actual epidemiology of FIs in primary care settings in high income country (HIC): one of the larger study covering the years between 1990 and 2021 exhibited an overall incidence and prevalence in HIC of 16,148 and 5,117 cases per 100,000, respectively, in 2021 even though this number is reported for adult and pediatric population. Considering only the pediatric population, the study described an incidence of 140,377,507 cases in the younger age group (5–9 years), but this data is not efficiently comparable because reflecting the global widespread of FIs, including invasive disease [4].

However, as previously reported [5], skin and mucosal FIs represent one of the leading causes of non-fatal morbidity worldwide based on the Global Burden of Disease [14, 15].

We also found that in almost half of the episodes, an antifungal medication was prescribed, and the majority was for the topical route. Most episodes were mild and did not require systemic medications, although systemic therapy is often needed for infections involving the hair follicles and the nails [12, 16]. Overall, we observed very few treatment failures: 1% for onychomycosis and less than 1% for skin and mucosal infections. These data are not fully comparable with the current literature due to different outcome measures, but most studies reported treatment success rates of around 95% for skin infections and 83% for onychomycosis with systemic antifungal therapy [17, 18].

Our study presented several limitations. We assessed prescriptions by the FPs, but it is possible that some cases were prescribed antifungal agents during a specialist dermatology visit, and the rate of treatment failure could therefore be higher than we estimated. It is also possible that some clinicians prescribed an antifungal, but recommended parents wait 1–2 days to evaluate the clinical course of the disease before giving any medication.

Additionally, we used treatment switching as a proxy for treatment failure, due to the nature of the dataset analyzed. This definition may only partially align with clinical evaluation and could also lead to an underestimation of treatment failure. Further, Joinpoint regression identifies changes in trends but does not establish causality; thus, observed inflection points should not be attributed to specific factors without further analysis.

Finally, we relied on clinical diagnosis, not symptoms, to identify an episode, and no microbiological confirmation was requested.

In conclusion, this large, population-based study provides an updated epidemiological overview of non-invasive FIs in the pediatric outpatient population. The findings highlight the considerable burden of *Candida*-associated infections in early childhood and underscore the importance of awareness among primary care providers. Improved diagnostic accuracy, preventive strategies, and evidence-based antifungal stewardship are essential to reduce morbidity and ensure appropriate management of these common but often overlooked infections.

Future studies could explore these results in relation to climate data. Temperature, humidity, and other environmental factors may affect fungal growth, adaptation, and survival.

The epidemiology of fungal infections (FIs) represents a major challenge in the understanding of fungal diseases and has been identified as a priority in the World Health Organization (WHO) research agenda for fungal pathogens because of their serious impact and clinical relevance [1].

Linking epidemiological trends with climatic conditions may provide valuable insights into the potential influence of climate change on fungal infections [19, 20]. However, in our study, the impact of environmental conditions and behavioral changes during the study period, including reduced healthcare-seeking behavior, fewer healthcare visits, and the intensification of infection control measures, is clearly illustrated by the marked decrease in the number of cases observed among the non-neonatal population during the COVID-19 pandemic (Figure S3). Nevertheless, most of the episodes involved newborns patients considering this population one of the most susceptible to the fungal infections [21].

## Electronic Supplementary Material

Below is the link to the electronic supplementary material.

Supplementary Material 1

## Data Availability

The data used in this study cannot be made available in the article, the Supplementary Materials, or in a public repository due to Italian data protection laws. The anonymized datasets generated during and/or analyzed during the current study can be provided on reasonable request, from the corresponding author, after written approval by the Internal Scientific Committee. Requests to access these datasets should be directed to Internal Scientific Committee (info@pedianet.it).

## Declarations

### Supplementary materials

Table S1: Complete list of fungal infections and adverse events codes considered. Table S2: Complete findings regarding the treatment prescription and the treatment outcome. Figure S3: Trend of fungal infection episodes stratified for the age of patients. Figure S4: Sankey diagram presents the antifungal treatment switching during the skin and mucocutaneous episodes.
