# Supplementary material for: Burden of non-invasive fungal infections and antifungal therapy in pediatric primary care settings: FUNGICARE Project
Source: Ital J Pediatr. 2026 May 26;52:114. doi: 10.1186/s13052-026-02277-8 (PMC13393752; doi:10.1186/s13052-026-02277-8)
Supplement: Supplementary file 1 — Supplementary Material 1 [file 13052_2026_2277_MOESM1_ESM.docx]

**Supplementary material**

**Table S1**. Complete list of fungal infections and adverse events codes considered

| Descriptive text | ICD9-CM |
| --- | --- |
| **Dermatophytosis (other and unspecified)** | **111.8, 111.9** |
| Aspergillosis | 117.3 |
| Other Systemic Mycoses | 117.7-**118** |
| Candida Infections  Candidiasis (all forms)  Neonatal candidiasis  Other forms of candidiasis | 112.0 – 112.9 771.7  112.81 – 112.89 |
| Diaper dermatitis | 691.0 |
| Otomycosis | 380.15 |
| Itch | 698.2, 698.8, 698.9 |
| Erythema/Skin rash | 695.1, 782.1 |
| Dermatitis/Eczema | 698.4, 692.3, 692.9, 782.8 |
| Edema | 782.3, 782.2 |
| Urticaria/ Skin vescicular | 708.0, 708.3, 708.9 |
| Pain at the application site | 682.0, 780.99 |
| Generic allergic reaction | 995.3 |
| Hypertrichosis | 704.1, 704.2 |
| Hypopigmentation | 709.0 |
| Skin atrophy | 701.8 |

**Table S2. Complete findings regarding the treatment prescription and the treatment outcome.**

*A – Onychomycosis*

|  | **Onychomycosis**  **N = 298*** |
| --- | --- |
| More than one prescription at the first consultation | 21 (7.0) |
| Treatment prolongation (same ATC prescribed later) | 72 (24.2) |
| Antifungal switch (different ATC code during the episode) | 3 (1.0) |
| Tioconazole → Ketoconazole (350 days) | 1 (33.3) |
| Fluconazole → Terbinafine (416 days) | 1 (33.3) |
| Itraconazole → Terbinafine (3 days) | 1 (33.3) |

ATC: Anatomical Therapeutic Code. *1 episode with ATC missing excluded

*B –* Skin and mucocutaneous mycoses

|  | **Skin and mucocutaneous mycoses**  **N = 11,622** |
| --- | --- |
| More than one prescription at the first consultation, n (%) | 1,086 (9.3) |
| Treatment prolongation (same ATC prescribed later) n (%) | 724 (6.2) |
| Antifungal switch (different antifungal ATC code during the episode) n (%) | 91 (0.7) |
| Switch time (in days), mean (sd) – median (Q1-Q3) | 22 (15) - 19 (11-32) |

ATC: Anatomical Therapeutic Code. **48 Patients with ATC missing excluded*


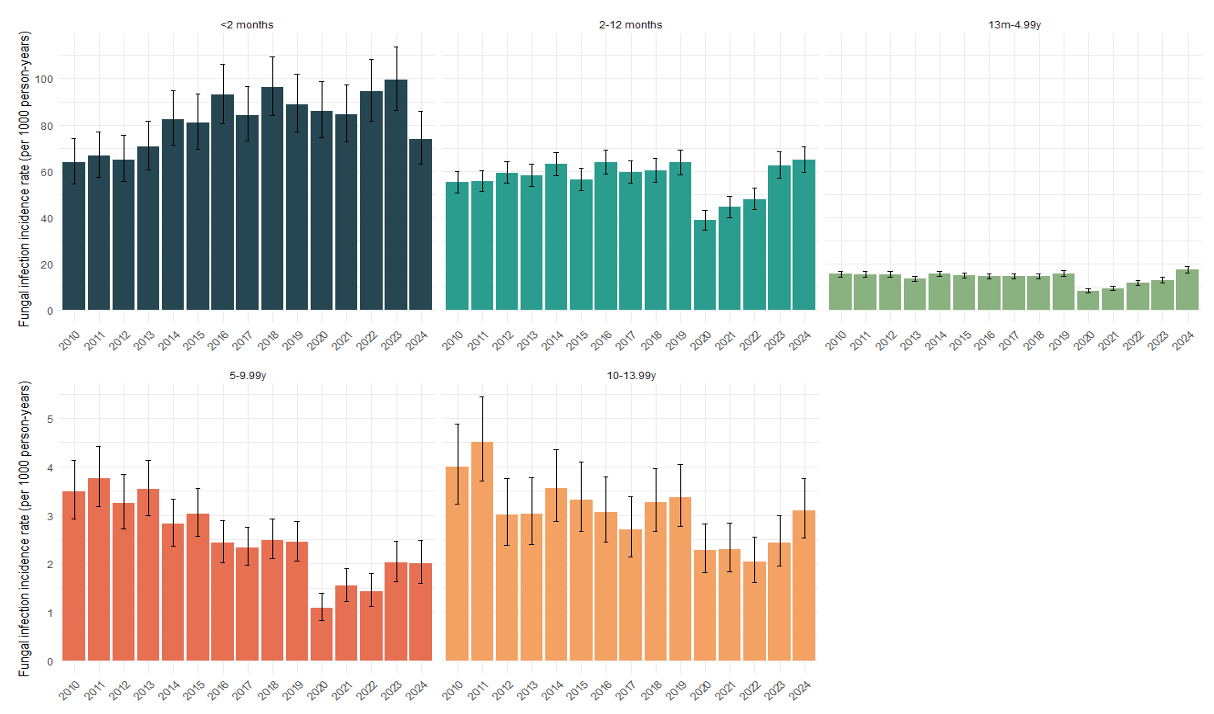


**Figure S3**. Annual incidence rate with 95% confidence intervals of fungal infection episodes stratified by age class.


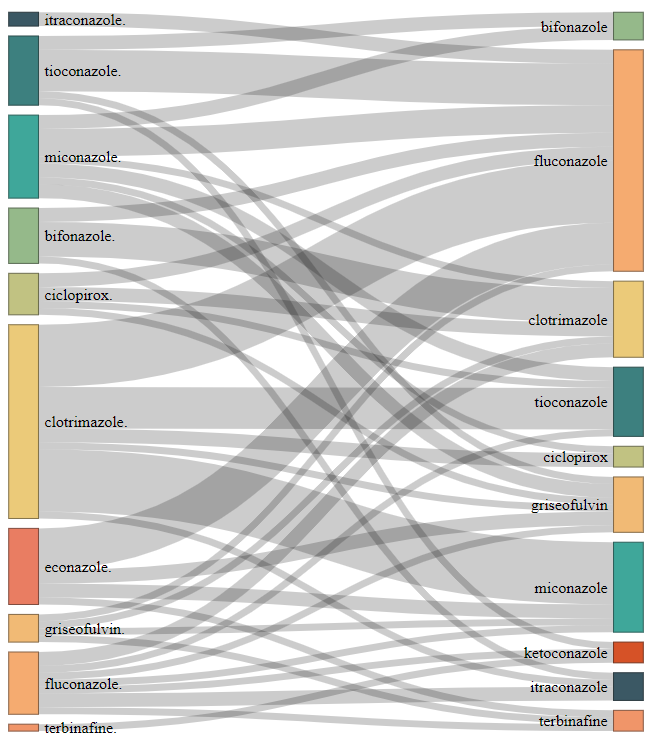


**Figure S4**. The Sankey diagram presents the antifungal treatment switching during the skin and mucocutaneous episodes (N = 91).
